# In-depth analysis of N_2_O fluxes in tropical forest soils of the Congo Basin combining isotope and functional gene analysis

**DOI:** 10.1038/s41396-021-01004-x

**Published:** 2021-05-25

**Authors:** Nora Gallarotti, Matti Barthel, Elizabeth Verhoeven, Engil Isadora Pujol Pereira, Marijn Bauters, Simon Baumgartner, Travis W. Drake, Pascal Boeckx, Joachim Mohn, Manon Longepierre, John Kalume Mugula, Isaac Ahanamungu Makelele, Landry Cizungu Ntaboba, Johan Six

**Affiliations:** 1 Department of Environmental Systems Science, Swiss Federal Institute of Technology, ETH Zurich, Zurich, Switzerland; 2 College of Agricultural Sciences, Oregon State University, Corvallis, OR USA; 3 School of Earth, Environmental, and Marine Sciences, University of Texas Rio Grande Valley, Edinburg, TX USA; 4 Isotope Bioscience Laboratory, Faculty of Bioscience Engineering, Ghent University, Ghent, Belgium; 5 Computational and Applied Vegetation Ecology Lab, Department of Environment, Ghent University, Ghent, Belgium; 6 Earth and Life Institute, Université Catholique de Louvain, Louvain, Belgium; 7 Laboratory for Air Pollution/Environmental Technology, Swiss Federal Laboratories of Materials Science and Technology, Empa Dubendorf, Switzerland; 8 Département de Biologie, Université Officielle de Bukavu, Bukavu, Democratic Republic of Congo; 9 Department of Green Chemistry and Technology, Ghent University, Ghent, Belgium; 10 Département d’ Agronomie, Université Catholique de Bukavu, Bukavu, Democratic Republic of Congo

**Keywords:** Microbiology, Biogeochemistry

## Abstract

Primary tropical forests generally exhibit large gaseous nitrogen (N) losses, occurring as nitric oxide (NO), nitrous oxide (N_2_O) or elemental nitrogen (N_2_). The release of N_2_O is of particular concern due to its high global warming potential and destruction of stratospheric ozone. Tropical forest soils are predicted to be among the largest natural sources of N_2_O; however, despite being the world’s second-largest rainforest, measurements of gaseous N-losses from forest soils of the Congo Basin are scarce. In addition, long-term studies investigating N_2_O fluxes from different forest ecosystem types (lowland and montane forests) are scarce. In this study we show that fluxes measured in the Congo Basin were lower than fluxes measured in the Neotropics, and in the tropical forests of Australia and South East Asia. In addition, we show that despite different climatic conditions, average annual N_2_O fluxes in the Congo Basin’s lowland forests (0.97 ± 0.53 kg N ha^−1^ year^−1^) were comparable to those in its montane forest (0.88 ± 0.97 kg N ha^−1^ year^−1^). Measurements of soil pore air N_2_O isotope data at multiple depths suggests that a microbial reduction of N_2_O to N_2_ within the soil may account for the observed low surface N_2_O fluxes and low soil pore N_2_O concentrations. The potential for microbial reduction is corroborated by a significant abundance and expression of the gene *nosZ* in soil samples from both study sites. Although isotopic and functional gene analyses indicate an enzymatic potential for complete denitrification, combined gaseous N-losses (N_2_O, N_2_) are unlikely to account for the missing N-sink in these forests. Other N-losses such as NO, N_2_ via Feammox or hydrological particulate organic nitrogen export could play an important role in soils of the Congo Basin and should be the focus of future research.

## Introduction

Primary tropical forests are generally considered nitrogen (N) rich, where N is cycled in excess of biological demands [1, 2]. High fire-derived N deposition rates combined with a downregulation of biological nitrogen fixation confirm the N richness of central African lowland forests [3, 4]. High N inputs are generally balanced by large N-losses resulting in an open N cycle. N-losses in tropical forests primarily occur through the leaching of nitrate (NO_3_^–^), dissolved and particulate organic nitrogen (DON and PON, respectively) to drainage waters, as well as through gaseous N-losses such as nitric oxide (NO), nitrous oxide (N_2_O) or elemental nitrogen (N_2_) resulting from denitrification. The lack of significant hydrological DON losses in the Congo Basin raises questions about the fate of the large N deposition loads [5] observed in this region. This imbalance suggests that gaseous N-losses may play a major role in the N-cycle of African tropical forests [6–8]. Of these gaseous N-losses, N_2_O is of high concern due to its significant global warming potential (265–298 CO_2_-equivalents) [9] and its destructive effect on stratospheric ozone levels [10]. As a result, research is currently directed towards understanding the mechanisms driving N_2_O emissions [9].

Natural soils play a central role in N_2_O production, representing 56% of atmospheric N_2_O sources [11], with tropical forest soils being the major natural producer [12]. The tropical forest of the Congo Basin, located primarily in the Democratic Republic of Congo (DRC), is the world’s second-largest tropical forest after the Amazon, covering 3.6 million km^2^. As such, it has been proposed to be one of the major natural sources of atmospheric N_2_O [13, 14]; and yet, despite its potentially high contribution to global N_2_O production, in situ studies of gaseous N-losses in the Congo Basin (and in African forests in general) are rare [12, 14, 15].

Within the Congo Basin, 44% of the land is covered with tropical lowland forests (hereafter referred to as “lowlands”) [16]. In addition to tropical lowlands, the Congo Basin contains tropical montane forests (hereafter referred to as “montane”) as part of the Albertine Rift region, which are situated in the east of the basin. Given large climatic differences between lowlands and montane forest, the Congo Basin provides an ideal location to investigate how abiotic and biotic factors may influence tropical N_2_O emissions.

**Fig. 1:**
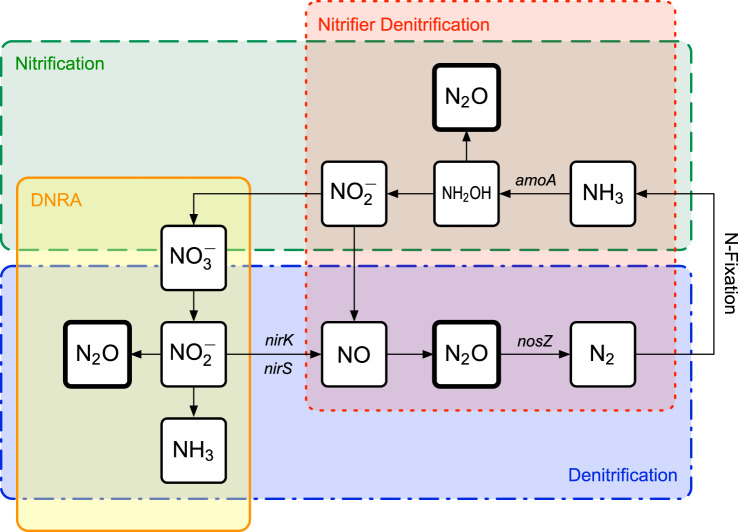
Main biotic N_2_O production and consumption pathways (nitrification, denitrification, nitrifier-denitrification, and dissimilatory nitrate reduction to ammonium DNRA) in soils. The marker genes involved in the biotic processes include: ammonia monooxygenase present in archaea (*amoA* *AOA*) and bacteria (*amoA* *AOB*), the two redundant nitrite reductases (*nirK*, *nirS*), and nitrous oxide reductase (*nosZ*).

In (forest) soils, N_2_O is produced by various biotic (Fig. 1) and abiotic processes, which can occur simultaneously within the same micro-ecosystem [17]. Biotic processes, such as nitrification and denitrification, are metabolic pathways controlled by microbial organisms (e.g. archaea, bacteria, and fungi). Nitrification refers to the conversion of ammonium (NH_3_) to nitrate (NO_3_^-^), whereas denitrification describes the stepwise reduction of nitrate (NO_3_^-^) to molecular nitrogen (N_2_) with nitrous oxide (N_2_O) as an obligatory intermediate [18]. Biotic nitrification and denitrification are considered to be the main N_2_O production pathways, and to account for ~70% of global N_2_O emissions [11, 19]. Nitrification can be incomplete, leading to nitrifier denitrification, where NO_2_^–^ is directly oxidized to NO followed by denitrification [20, 21]. A further contributor to soil N_2_O production is dissimilatory nitrate reduction to ammonia (DNRA), in which NO_3_^–^ is reduced to NO_2_^–^, and then further reduced to ammonium (NH_4_^+^) [18]. Although there is evidence that N_2_O is produced as a byproduct of DNRA, the actual contribution of DNRA to total N_2_O formation remains uncertain [22]. The ability to perform DNRA is widespread and performed by a diverse group of bacteria and fungi of the Ascomycota phylum [18, 23, 24].

The abundance and expression of key functional marker genes encoding for enzymes involved in various steps of the N-cycle can be used to assess biotic N_2_O production and reduction pathways. For example, nitrifiers can be analyzed by targeting the *amoA* gene, which encodes for the ammonia monooxygenase present in archaea (*amoA* *AOA*) and bacteria (*amoA* *AOB*). This enzyme catalyzes the first step of nitrification: the oxidation of NH_3_ into hydroxylamine (NH_2_OH) [25–27]. Nitrifying microorganisms are a phylogenetically conserved group of archaea and bacteria [28]. Denitrifiers can be analyzed by targeting the two redundant nitrite reductase genes *nirK* and *nirS*, which encode enzymes involved in the second step of denitrification: the reduction of NO_2_^–^ into NO, an important precursor of N_2_O [29–31]. Denitrifiers can also be studied by targeting *nosZ*, which encodes the nitrous oxide reductase involved in the last step of denitrification: the reduction of N_2_O into N_2_. Two phylogenetically distinct clades of *nosZ* are known: *nosZ* type I and type II [32–34]. The *nosZ* gene is frequently used as a marker to measure the occurrence of N_2_O reduction [35]. Indeed, microbes are often referred to as N_2_O-reducers if they harbor the N_2_O reductase gene without possessing other genes involved in the previous denitrification steps [36]. Present in over 60 genera, denitrifying bacteria are widely distributed over different taxa and metabolic groups [37]. In addition to bacteria, fungi, belonging mainly to the phylum Ascomycota [38], are also capable of denitrification. The significance of abiotic N_2_O production pathways such as the chemical decomposition of hydroxylamine [39, 40] and chemical decomposition of soil nitrite [39] is not yet well understood, since distinguishing abiotic from biotic processes under field conditions is methodologically challenging.

Whether gaseous N escapes the system as toxic NO, as global warming inducing N_2_O, or as inert N_2_, is determined by environmental factors including oxygen availability, inorganic nitrogen substrates (NH_3_, NO_2_^–^, NO_3_^–^), available organic carbon and micronutrients, as well as soil pH, temperature, and the composition of the soil’s microbial community [21, 22, 41]. Indeed, many studies have relied on the quantification of these environmental factors and substrate availability to infer pathways of N_2_O production.

Apart from monitoring soil conditions and substrates or analyzing soil marker genes, the isotopic composition of N and O in N_2_O (*δ*^15^N and *δ*^18^O) can be used to identify the active microbial N_2_O pathways. This approach relies on the general fractionation in favor of lighter isotopes (^14^N and ^16^O) rather than heavier isotopes (^15^N and ^18^O) during biochemical reactions. N_2_O produced during nitrification is generally more depleted in ^15^N and ^18^O relative to the substrate than N_2_O produced during denitrification, allowing these two processes to be distinguished [22]. This difference between the product and substrate isotope values is referred to as the apparent isotope effect (ε) or net isotope effect if spanning multiple intermediary steps (η). Limitations of this method, however, arise from the dependency on accurate knowledge of the substrate’s *δ*^15^N and *δ*^18^O (i.e., in NH_4_^+^ and NO_2_^–^, NO_3_^–^), which are subject to large temporal and spatial variations and are laborious to quantify [42]. Additionally, limited data is available for fractionation factors of some processes involved in N_2_O production (i.e., DNRA) and their dependency on the specific microbial strains [17, 43].

Advances in isotopic ratio mass spectrometry and laser spectrometry have yielded new insights into the intra-molecular distribution of ^15^N within the asymmetric N_2_O molecule. The site preference (*SP*) describes the difference between *δ*^15^N at the central position (*α*) compared to the terminal N atom (*β*) (*SP* = *δ*^15^N^*α*^ – *δ*^15^N^*β*^). A recent review showed that N_2_O produced via nitrification (NH_4_^+^ oxidation by *amoA AOA* and *amoA AOB*) exhibits an ~30‰ higher *SP* value (28.6 ± 4.8‰) than via denitrification (1.6‰ ±3.0‰) [42]. However, the *SP* of N_2_O produced from nitrification overlaps with that of fungal denitrification while, similarly, the *SP* of N_2_O produced from denitrification overlaps with that from nitrifier denitrification. Nonetheless, the large separation in *SP* values is a powerful tool to distinguish between these two process pools (i.e., nitrification/fungal denitrification and denitrification/nitrifier denitrification). Furthermore, unlike *δ*^15^N (as *δ*^15^N^bulk^ = $\frac{1}{2}$ (*δ*^15^N^*α*^ + *δ*^15^N^*β*^)) and *δ*^18^O, *SP* is independent of the isotopic signature of the substrates, allowing a more robust distinction of N_2_O produced by different production processes [44, 45]. However, it is not feasible to use a single indicator, i.e., *SP*, to separate multiple processes with overlapping signatures and variations between different microbial strains, which are yet to be fully understood [42, 46, 47]. Co-dependence between different isotopic tracers, e.g. the simultaneous enrichment of *δ*^15^N^bulk^, *δ*^18^O, and *SP* in N_2_O, remaining after partial reduction to N_2_, is a way to cope with the complexity of N_2_O production pathways [48, 49]. An innovative alternative approach to overcome these limitations and more-accurately identify relative contributions of different N_2_O production and consumption processes, is the combination of isotopic signatures with the abundance and expression of functional genes that encode enzymes involved in N_2_O production and consumption processes [17, 22].

Thus, we combined natural isotopic signatures of N_2_O in soil pore air with qPCR-derived gene abundance and expression data of nitrifying and denitrifying gene-bearing communities of a lowland and montane forest soil in the Congo Basin in order to understand the underlying mechanisms driving N_2_O soil fluxes in these two different tropical forest ecosystem types. We hypothesize that the general magnitude of N_2_O emissions across all forest soils of the Congo Basin is similarly high as compared to other tropical forest soils. If supported, this finding would emphasize the large potential of the Congo Basin to contribute significantly to global N_2_O emissions. We further hypothesize that N_2_O emissions are higher in the lowlands compared to the montane forest due to a more open N cycle that is induced by favorable climatic conditions (higher temperatures) that facilitate the decomposition of organic matter. If supported, this finding would point towards a low bioavailable N retention in lowland forests.

## Materials and methods

### Study areas

Two sampling campaigns were conducted in the Democratic Republic of Congo (DRC) between May 2017 and March 2019 at two study sites: The Yoko Forest Reserve (lowlands) and the Kahuzi-Biéga National Park (montane).

The Yoko forest reserve covers an area of ~70 km^2^ and is located south of the city of Kisangani in the province of Tshopo (0.30°S, 25.3°E). Characterized as a tropical primary lowland forest (413 m a.s.l), Yoko’s forests consist of areas with a monodominant presence of *Gilbertiodendron dewevrei* that cover about 70% of the basal area with the remaining area comprised of mixed vegetation of about 80 species per hectare and a canopy height of up to 40 m [50]. Our sampling site was established in a mixed forest, where soils are characterized as Ferralsols, commonly found throughout the entire Congo Basin [51]. The mean air temperature is ~25 °C and shows little temporal variation. Observed mean annual precipitation in Yoko is 1800 mm and follows a seasonal pattern with two distinctive peaks of heavy rainfall in April and October [5].

The Kahuzi–Biéga National Park is located in the Eastern part of the DRC in the South-Kivu province and covers an area of ~6000 km^2^. Our site was established near the park entrance at an altitude of 2126 m a.s.l (2.31°S, 28.76°E), where the vegetation is dominated by a tropical mixed montane forest [52]. Soils in the Kahuzi-Biéga National Park are Ferralsols/Acrisols, with relatively higher sand and silt contents compared to the lowland forest [5]. The mean air temperature in the montane forest is 20 °C, showing little temporal variation. Annual precipitation ranges between 1500 and 2000 mm and follows a seasonal pattern, with the wet season occurring from September to May, followed by a short dry period from June to August [5].

Physicochemical parameters and the abundance of micronutrients of the sampled soils are summarized in Table 1 and Table S1, respectively.

**Table 1:** Texture and pH at the two forest ecosystem types (lowlands and montane).

Forest site	Depth (cm)	Clay (%)	Silt (%)	Sand (%)	pH
Lowlands	0–15	3.1	11.0	85.9	3.93 ± 0.02
	15–30	6.2	11.3	82.6	4.01 ± 0.04
Montane	0–15	6.7	47.1	46.2	6.08 ± 0.00
	15–30	9.7	70.0	20.3	5.25 ± 0.42

### Physicochemical soil characteristics

Composite soil samples at two depths (surface layer: 0–5 cm; subsurface layer: 5–20 cm) were collected at each site during the first sampling campaign (lowlands: 7th, 17th, 27th of June 2017; montane forest: 5th of May 2017). One soil sample represents a composition of three soil cores collected in the vicinity of each flux chamber. In addition to the soil samples, ~20–25 random fresh leaves were collected as composite sample from trees surrounding the flux chambers at each site. In a similar manner, dead leaves were collected from the forest floor at each site. After collection, soil, leaf, and litter samples were oven dried for 2 days at 60 °C and transported back to ETH Zurich, where all further analyses were performed.

Soil pH was assessed following the protocol described in Sparks et al. 1996 [53] using H_2_O as a solute. The pH was measured using a 1000 L pH meter (VWR, Radnor, PA, USA).

Soil texture was assessed by the addition of 4 mL of 10% Na-hexametaphosphate to 0.8 g of sandy soil samples and to 0.08 g of loamy soil samples. After 4 h of shaking and 1 min of sonication, the samples were analyzed in a particle size analyzer (LS 13 320, Beckman Coulter, IN, USA). The United States Department of Agriculture (USDA) classification was used to determine the soil texture class.

Wet digestion was performed to measure total P, K, Ca, Mg, S, Na, Zn, and Fe using aqua regia solution. Soil samples of 1.0 ± 0.001 g weighted in digestion tubes were mixed with 2 mL of nanopore water, 2 mL of nitric acid (HNO3 70%), and 6 mL of hydrochloric acid (HCI 37%) and placed in a digestion block at 120 °C for 90 min covered with watchglasses. After cooling, the solution was brought to 50 mL volume with nanopore water and filtered (Watchman no 41) to centrifuge tubes. The digests were diluted (1:10) and analyzed by Inductively Coupled Plasma Optical Emission Spectrometry (ICP OES, Agilent).

Dried soil and plant material were milled using a ball mill. The C/N ratio of the soil, leaves, and litter was determined with a C/N analyzer (CHN 628, Leco Corporation, St. Joseph, MI, USA).

The *δ*^15^N values of the soil, leaf, and litter were measured with an elemental analyzer (Flash EA) coupled to a Delta^plus^XP isotope ratio mass spectrometer (IRMS) via a 6-port valve [54] and a ConFlo III interface (both Finnigan MAT, Bremen, Germany; [55]). The measurement sequence of samples, blanks, and standards followed the scheme described in Werner et al. 2001 [55].

### Water-filled pore space (WFPS)

At each site, soil moisture probes (PR2/6 Profile Probe, Delta-T Devices, Cambridge, UK) and sensors (ECH_2_O-EC-5 Probe, Meter Environment, Munich, Germany) were installed and recorded with an interval of four hours. Probes were installed during a second sampling campaign from the 16^th^ of March 2018 until the 17^th^ of March 2019 at the lowland site and from 26th of March until the 16th of October 2018 at the montane forest site. In order to compare the measured volumetric water content (VWC) between sites, the water-filled pore space (WFPS) in the units of % was calculated as

$$WFPS = 100\,\frac{{VWC}}{{1 - \frac{{BD}}{{PD}}}},$$

where BD represents the soil bulk density provided by Bauters et al. 2019 [5] for the montane forest soil (0.75 ± 0.17 g cm^–3^) and the lowland forest soil (1.35 ± 0.06 g cm^–3^) and where PD represents the mineral particle density (2.65 g cm^–3^).

### N_2_O soil surface fluxes

Soil surface N_2_O fluxes were measured using the manual static chamber technique. Air samples were collected in static PVC (Polyvinylchloride) chambers with a volume of ~17 L and an area of 0.07 m^2^. The chamber was equipped with airtight lids, thermocouples, sampling ports, and vent tubes to reduce pressure interferences. At each site (lowlands and montane), five chambers were placed on the forest floor. Flux measurements were conducted during the second sampling campaign from April 17th 2018 until March 17th 2019. During the study period, each chamber was sampled once every 2 weeks at midday. For sampling, chambers were closed for 1 h and headspace samples were collected at intervals of 20 min (*t*_1_ = 0, *t*_2_ = 20, *t*_3_ = 40, and *t*_4_ = 60 min) using a 20 mL syringe. Prior to sample collection, the syringe was flushed once with air from the chamber headspace to homogenize and mix air within the chamber. At each time interval, the 20 mL air sample was collected and injected into pre-evacuated 12 mL vials (Labco, Lampeter, Wales, UK), which were additionally sealed with silicone (Dow Corning 734) prior to sampling to ensure air tightness. The temperature inside the chamber was measured at each time interval using a thermocouple (Type T, Omega Engineering, Stamford, CT, USA). Gas samples were transported back to the laboratory at ETH Zurich, where N_2_O concentrations were measured using a gas chromatograph equipped with an electron capture detector (456-GC, Scion Instruments, Livingston, WLO, UK).

N_2_O flux rates were calculated from the concentration change over time, using a linear regression model for the four consecutive gas measurements during a measurement cycle. The slope of the linear regression line given in [mol L^–1^ s^–1^] was converted into a flux [mol m^–2^ s^–1^] by accounting for headspace volume V [L] and chamber area [m^2^].

### N_2_O subsurface pore air concentration

In addition to the N_2_O surface flux samples, air samples from the soil pore space were collected at each site using passive diffusion probes installed at three different depths (20, 50, and 100 cm) described in detail in Verhoeven et al. 2019 [56]. A 10 cm diffusion cell was attached to the end of a probe shaft with a length of 50–100 cm. Each probe was equipped with two sampling ports in order to attach one pre-evacuated serum crimp vial (110 mL) above ground for N_2_O stable isotope analysis and one pre-evacuated Labco vial (12 mL) for N_2_O concentration measurements. Samples were collected passively during the second sampling campaign over a time period of 2 weeks, i.e., the vials were left attached to the sample outlets of the probes in order to allow equilibration with soil air. Subsequently, vials were stored and transported back to ETH Zurich, where N_2_O concentrations were measured with a GC (N_2_O soil surface fluxes section) and N_2_O isotopic composition using IRMS as detailed in N_2_O isotopic analysis section.

### N_2_O isotopic analysis

*δ*^15^N^bulk^, *δ*^18^O, and *SP* of the soil pore air N_2_O samples was measured using a gas preparation unit (Trace Gas, Elementar, MCR, UK) coupled to an IRMS (IsoPrime100, Elementar, MCR, UK) at ETH Zurich. IRMS calibration was done using three sets of two working standards (∼3 ppm N_2_O mixed in synthetic air) with different isotopic compositions (δ^15^N^α^ = 0.95 ± 0.12‰ and 34.45 ± 0.18‰; δ^15^N^β^ = 2.57 ± 0.09‰ and 35.98 ± 0.22‰; δ^18^O = 39.74 ± 0.05‰ and 38.53 ± 0.11‰). These standards were analyzed at the Swiss Federal Laboratories for Materials Science and Technology (EMPA) using TREX-QCLAS versus standards with assigned δ-values by Tokyo Institute of Technology (Mohn et al. 2014 [57]). Three sets of standards were included with a batch of 20 samples, one at the start, middle, and at the end of each run. Sample peak ratios were initially reported against an N_2_O reference gas peak (100% N_2_O, Carbagas, Gümligen, Switzerland) and were subsequently corrected for drift and span using the working standards. Instrument linearity and stability were frequently checked by injection of 10 reference gas pulses of either varying or identical height, respectively, with accepted levels of < 0.03‰ nA^−1^. Since instrument linearity could only be achieved for either N_2_O or NO, the instrument had been tuned for the former and δ^15^N^α^ subsequently corrected using a second set of the two working standards within each run, which were diluted to atmospheric concentrations. Dilution from the main working standards at ~3 ppm N_2_O to ~330 ppb N_2_O and subsequent gas cylinder filling was performed at EMPA. The overall average deviation from true value for the diluted standards across all IRMS measurement runs was −0.45‰, 0.52‰, and −0.56‰ for δ^15^N^Bulk^, δ^15^N^α,^ and δ^18^O, respectively after calibration based on non-diluted standards.

^15^*N*/^14^*N* isotope ratios are reported relative to the international isotope ratio scale AIR-N2 while ^18^*O*/^16^*O* are reported versus Vienna-Standard Mean Ocean Water (V-SMOW). Relative differences are given using the delta notation (*δ*) given in ‰: $\delta ^Z{\mathrm{X}} = ( {\frac{{R_{sample}}}{{R_{reference}}} - 1} ),$ where *R* refers to the molar ratio of ^15^N/^14^N or ^18^O/^16^O and ^Z^X refers to the heavy stable isotope Z of the element X.

### Gene abundance and expression

Composite soil samples were collected at each site during the first sampling campaign (lowlands: 7th, 17th, 27th of June 2017; montane forest: 5th of May 2017). During sampling, three soil cores were collected and composited by depth (surface layer: 0–5 cm; subsurface layer: 5–20 cm). A subsample of 2 g was added to a 6 mL solution of LifeGuard Soil Preservation Kit (Qiagen, Hilden, NW, Germany). Shortly after sampling, the solutions were frozen and transported back to the lab at ETH Zurich for analyses. Due to a low performance of the LifeGuard solution leading to degraded DNA, additional seasonal soil samples collected in March 2018, July 2018, October 2018, and January 2019 at both sampling sites were unable to yield reliable high-quality results for N-cycling marker gene analyses.

RNA and DNA were extracted using commercial kits following the manufacturer’s protocol (RNeasy PowerSoil Total RNA Kit and RNeasy PowerSoil DNA Elution Kit, Qiagen, NW, Germany). Additionally, clean-up kits removed interfering humic substances in the RNA and DNA samples (RNeasy PowerClean Pro Cleanup Kit and DNeasy PowerClean Pro Cleanup Kit, Qiagen, Hilden, NW, Germany). DNA and RNA concentrations and quality were quantified with a Qubit fluorometer (Thermo Fisher Scientific, Waltham, MA, USA) and a NanoDrop spectrophotometer (Thermo Fisher Scientific, Waltham, MA, USA), respectively. The Invitrogen SuperScript^*TM*^ IV *V ILO*^*TM*^ Master Mix (ThermoFisher Scientific, Waltham, MA, USA) synthesized cDNA from the RNA samples. Prior to the reverse transcriptase, genomic DNA (gDNA) contamination from the template RNA was removed using ezDNase (Thermo Fisher Scientific, Waltham, MA, USA). DNA and cDNA were diluted with ddH_2_O to optimize the copy numbers within the detection limits of the real-time quantitative PCR measuring device. The DNA and cDNA extracts were frozen and stored for one week before application.

The abundance of nitrifying and denitrifying gene-bearing communities was assessed by quantitative real-time polymerase chain reaction (hereafter “RT-qPCR”) targeting the universal ribosomal gene *16* *S* [58], archaea and bacterial ammonia monooxygenase genes (*amoA AOA, amoA AOB* [25–27]), nitrite reductase genes (*nirK, nirS* [29–31]) and the nitrous oxide reductase gene *nosZ* [35].

Gene copy and transcript numbers were quantified by applying the following protocol. A master mix consisting of a gene-specific dye, primers (forward and reverse), and ddH_2_O was prepared. After preparation, the master mix was applied to a well-plate (96 or 384 wells) followed by the addition of diluted DNA or cDNA extracts in triplicates. The Master Mix composition and diluted DNA/cDNA extracts used for the real-time qPCR are provided in Table S2. Quantification of the genes *16* *S*, *nirK*, *nirS,* and *nosZ* was performed on a 7500 FAST RT-PCR System (Applied Biosystems, Foster City, CA, USA) whereas *amoA AOA* and *amoA AOB* were quantified using a Lightcycler 480 (Roche, BL, Switzerland). Primers and qPCR conditions used for RT-qPCR are summarized in Table S3. Standard curves were generated using a triplicate of 10-fold serial dilutions from 10^4^ to 10^9^ (*16* *S*); from 10^2^ to 10^7^ (*nosZ*, *nirK*, *nirS*); from 10 to 10^6^ (*amoA AOA*); and from 10^3^ to 10^8^ (*amoA AOB*) of plasmids containing the particular gene sequences. The sealed plate was centrifuged for 1 min at 2000×*g* before being inserted into the RT-qPCR device.

Genetic data produced and analyzed in this paper were generated in collaboration with the Genetic Diversity Centre (GDC) at ETH Zurich.

### Data analysis

Gene copy and transcript numbers per gram dry soil of the N-cycling marker genes were log transformed and tested for normality using the Shapiro-Wilk test. This test revealed that 90% of the data was log-normal distributed. Gene data was tested for outliers using the Interquartile Range (hereafter IQR). Points lying more than 1.5 IQRs from the mean were identified as outliers and removed from further data analysis (87 of 1505 data points were removed). The contributions of the two factors “Forest Type” (*n* = 2; lowlands, montane) and “Depth” (*n* = 2; surface layer, subsurface layer) were evaluated using one-way and two-way analyses of variance (ANOVA). We conducted a post-hoc analysis using Tukey’s Honest Significant Differences test (Tukey’s HSD). Each possible pairing of the two factors (“Forest Type,” “Depth”) were tested, with the level of significance for both ANOVA and Tukey’s HSD test set at *p* < 0.05. The pairwise Tukey’s HSD test was performed using the pairwise_tukeyhsd function from the statsmodels library of the python programming language.

## Results

### Physicochemical soil characteristics

Soils in the lowlands were on average much sandier than the montane site (84.3 and 33.3% sand across depths, respectively), and 1.7 pH units more acidic (Table 1). Furthermore, soils in the lowlands were generally poorer in micronutrients compared to the montane forest including manganese, iron, and sulfur (Table S1).

**Fig. 2:**
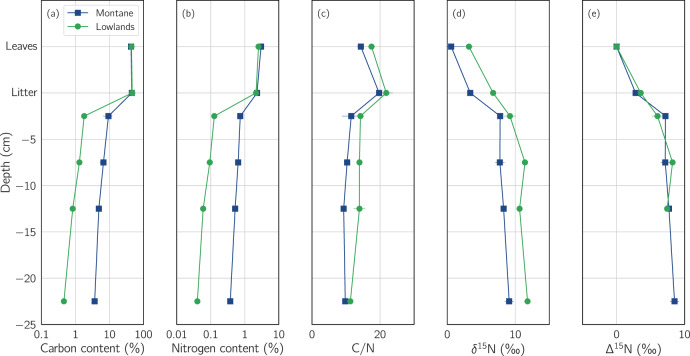
Physicochemical characteristics of fresh leaves, leaf litter, and soil at various depths (*−*5 to *−*25 cm). **a**, **b** The total C and total N content [%], respectively; **c** the C/N ratio; **d** isotopic signature δ^15^N [‰]; **e** difference between the isotopic signature (δ^15^N) of the source (leaves) and the sinks (litter and deeper soil layers) ∆^15^N. Where multiple measurements were available at a specific depth, each plot marker represents the mean of these measurements, and the standard deviation of these measurements is shown as a horizontal line.

Total carbon content in the leaves and litter was similar at both sites (Fig. 2) and averaged 44.7 ± 1.3%. Averaged across all soil layers, the montane forest showed a 5.6-times larger C content (6.10 ± 2.45%) compared to the lowlands (1.10 ± 0.58%).

Total N content in the leaves was higher at the montane site (3.00 ± 0.20%) compared to the lowlands (2.53 ± 0.14%). Nitrogen content in the soil of the montane forest (0.57 ± 0.16%) was 7.6 times larger than in the lowlands (0.08 ± 0.04%). Both carbon and nitrogen content showed a general decrease with depth at both sites.

The C/N ratio for both leaves and soil was slightly larger in the lowlands (leaves: 17.50 ± 1.08, soil: 13.36 ± 1.57) compared to the montane forest (leaves: 14.35 ± 1.01, soil: 10.12 ± 1.27).

Leaf and soil δ^15^N were higher at the lowland site (3.19 ± 0.67‰ and 10.76 ± 1.14‰, respectively) than at the montane site (0.58 ± 2.34‰ and 8.22 ± 0.73‰, respectively), and displayed an increasing soil ^15^N enrichment with depth at both sites.

The ∆^15^N describes the difference between the isotopic signature (δ^15^N) of the leaves and their decomposition products in the litter and at different soil layers, and was found to be very similar at both sites.

### Water-filled pore space (WFPS)

**Fig. 3:**
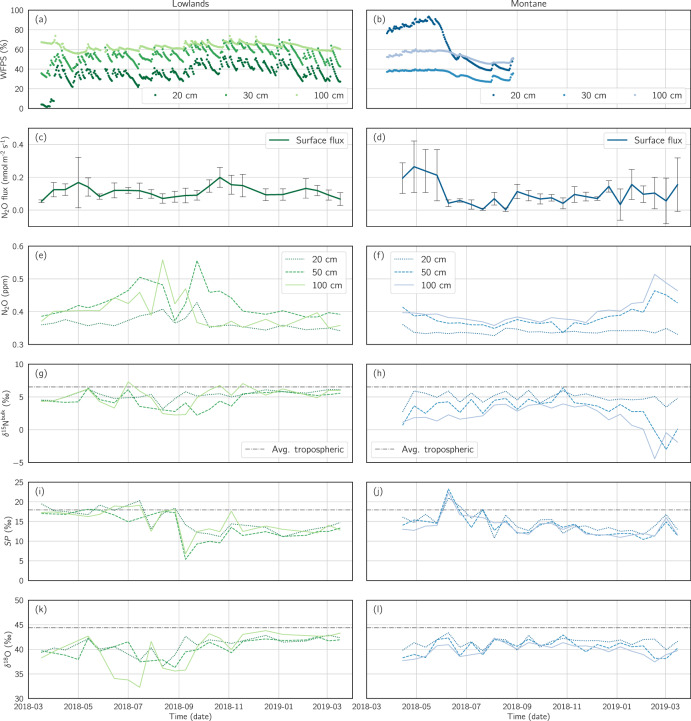
Time series data from the two forest ecosystem types (lowlands, montane). **a**, **b** Water-filled pore space (WFPS) [%]; **c**, **d** Surface N_2_O flux [nmol m^−2^ s^−1^] as measured in the PVC flux chambers; the data series shows the mean measurement across all flux chambers, while the vertical gray bars show the standard deviation; **e**, **f** N_2_O subsurface concentration [ppm]; **g**, **h** Subsurface N_2_O isotopic signature δ^15^N^bulk^ [‰]; **i**, **j** *SP* of subsurface N_2_O [‰]; **k**, **l** Subsurface N_2_O isotopic signature δ^18^O. Measurements were taken fortnightly (except WFPS, which was measured every 4 h) from March 16th 2018 until March 17th 2019 at the lowlands (green) and from March 26th 2018 until March 17th 2019 at the montane site (blue), respectively. Technical device malfunction at the montane forest site resulted in an incomplete timeseries for WFPS, which ended on August 27th 2018. Average isotopic composition of tropospheric N_2_O (δ^15^N^bulk^, *SP*, δ^18^O [114]) is illustrated as a gray dashed line (**g**–**l**).

Average annual WFPS in the montane upper 20 cm soil (64.3 ± 20.1%) was 78% larger than in the lowlands (36.2 ± 9.8%). In the wet-season of the montane forest (mid-March until late May), WFPS reached a maximum of 93.4% at a depth of 20 cm, and 39.9% at a depth of 30 cm. In the dry season of the montane forest, WFPS decreased to a minimum of 38.8% at a depth of 20 cm and 27.0% at 30 cm. Such a pronounced seasonal difference was not noticed at 100 cm depth, with WFPS values ranging from a minimum of 45.5% in the dry season to a maximum of 60.2% in the wet season (Fig. 3b). No seasonal effects were apparent in the lowlands (Fig. 3a), and weekly temporal variations were attributable to rain events. Data gaps in the WFPS measurements of the montane forest (Fig. 3b) were due to technical sensor malfunctions.

### N_2_O surface fluxes, subsurface concentrations, and isotopic signature

Average annual N_2_O fluxes in the lowlands (0.97 ± 0.53 kg N ha^−1^ year^−1^) were comparable to those in the montane forest (0.88 ± 0.97 kg N ha^−1^ year^−1^). Fluxes showed little seasonal variability in the lowlands (Fig. 3c); however, N_2_O fluxes in the montane forest (Fig. 3d) were much higher at the end of the wet season (1.94 ± 1.24 kg N ha^−1^ year^−1^, from mid-March until the end of May) than during the dry season (0.53 ± 0.53 kg N ha^−1^ year^−1^, June to September).

Observed annual N_2_O concentrations at a depth of 20 cm (Fig. 3e, f) were similar in the lowlands (0.37 ± 0.02 ppm) compared to those observed at the montane forest site (0.34 ± 0.01 ppm), and showed little seasonal influence. In contrast, seasonal effects were observed at 50 cm and 100 cm depths at both sites. At the lowland site, N_2_O concentrations remained relatively constant between November and May (0.40 ± 0.016 ppm at 50 cm, and 0.38 ± 0.02 ppm at 100 cm), and showed increased mean and variation from June to October (0.46 ± 0.048 ppm at 50 cm and 0.42 ± 0.06 ppm at 100 cm). At the montane site an opposite seasonal effect was observed, with N_2_O concentrations remaining relatively constant from April until December (0.37 ± 0.016 ppm at 50 cm and 0.38 ± 0.013 ppm at 100 cm) and showing increased mean and variation from January until March (0.42 ± 0.028 ppm at 50 cm and 0.45 ± 0.04 ppm at 100 cm).

Similar seasonal trends were visible in *δ*^15^N^bulk^ data from both sites, at both 50 and 100 cm depths. At the lowland site, *δ*^15^N^bulk^ remained relatively constant between November and May (5.07 ± 0.76‰ at 50 cm, and 5.44 ± 0.88‰ at 100 cm), and showed decreased mean and increased variation from June to October (3.71 ± 1.09‰ at 50 cm, and 4.61 ± 1.83‰ at 100 cm). At the montane site *δ*^15^N^bulk^ remained relatively constant from April until December (3.77 ± 1.14‰ at 50 cm, and 2.75 ± 0.98‰ at 100 cm) and showed decreased mean and increased variation from January until March (1.05 ± 2.38‰ at 50 cm, and −0.61 ± 2.16‰ at 100 cm).

*SP* and *δ*^18^O did not differ significantly between the two sites and showed little variation across various depths (Fig. 3g–l) or seasons, with an average *SP* measurement of 14.33 ± 2.90‰ across both sites, all depths, and throughout the year; and an average *δ*^18^O measurement of 40.44 ± 2.00‰ across both sites, all depths, and throughout the year.

### Gene abundance and expression

**Fig. 4:**
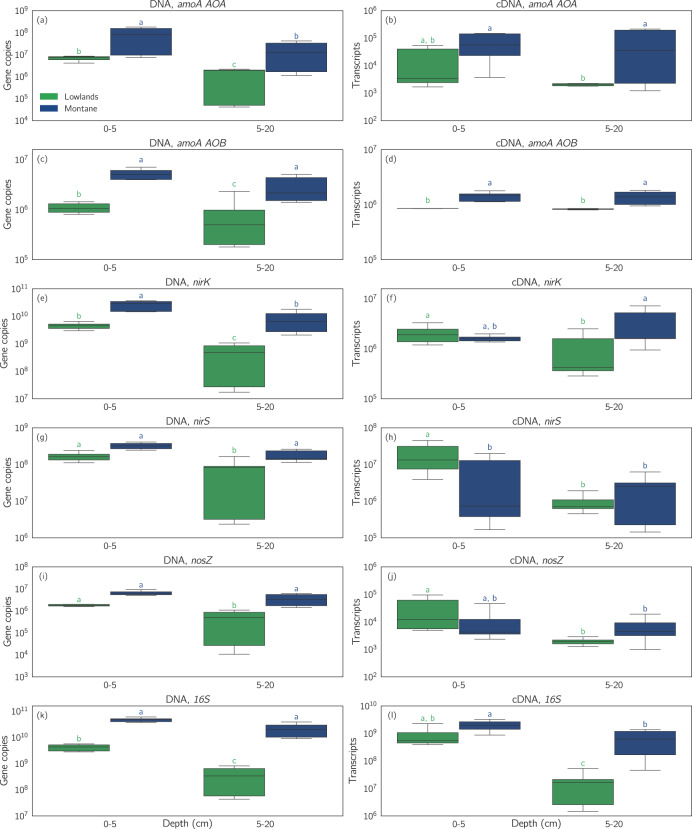
Gene abundance (DNA) [gene copies] and gene expression (cDNA) [transcripts] of the marker genes *amoA AOA*, *amoA AOB*, *nirK*, *nirS*, *nosZ,* and *16* *S* per gram dry soil. Composite soil samples were collected at each site during the first sampling campaign (lowlands: 7th, 17th, 27th of June 2017; montane forest: 5th of May 2017). During sampling, three soil cores were collected and composited by depth (surface layer: 0–5 cm; subsurface layer: 5–20 cm). The Tukey letters determined by the post hoc analyses (Tukey’s Honest Significant Differences test) are provided at the top of each box. The level of significance was set at *p* < 0.05.

Figure 4 shows the site-, and depth-specific gene abundance (DNA) [gene copies] and expression (cDNA) [gene transcripts] of the N-cycling marker genes *amoA AOA*, *amoA AOB*, *nosZ*, *nirK*, *nirS*, and *16* *S* per gram dry soil.

### Nitrifiers

Significant differences between the expression of *amoA AOA* could be observed in the subsurface layer (5–20 cm) with average *amoA AOA* expression being ~1.6 orders of magnitude larger in the montane forest (8.6*×*10^4^ ± 9.2*×*10^4^ transcripts) compared to the lowlands (2.0*×*10^3^ ± 2.0*×*10^2^ transcripts; Fig. 4a, b). In contrast to *amoA AOA*, significant differences between the two sites in *amoA AOB* abundance and expression were already apparent in the surface layer (0–5 cm). On average, *amoA AOB* was ~1.7 times more expressed in the subsurface of the montane forest (1.4*×*10^6^ ± 3.3*×*10^5^ transcripts) compared to the lowlands (8.3*×*10^5^ ± 1.7*×*10^4^ transcripts; Fig. 4c, d).

### Denitrifiers

Gene expression of *nirK* in the subsurface of the montane forest was ~3.3 times larger (3.3*×*10^6^ ± 2.6*×*10^6^ transcripts) compared to the lowlands (10.0*×*10^5^ ± 9.2*×*10^5^ transcripts; Fig. 4e, f).

In contrast to *nirK*, *nirS* expression did not differ significantly between the two study sites in the subsurface (Fig. 4e–h). Mean *nirS* expression in the subsurface was ~2.1*×*10^6^ ± 1.8*×*10^6^ transcripts across both forest types. Differences in gene expression of *nirK* and *nirS* between the surface and subsurface were only apparent in the lowlands, where *nirK* was ~2 times more expressed in the surface layer (2.0*×*10^6^ ± 8.2*×*10^5^ transcripts) compared to the subsurface layer (10.0*×*10^5^ ± 9.2*×*10^5^ transcripts). As for *nirS*, this was even more pronounced with 20 times larger *nirS* expression in the surface layer (2.0*×*10^7^ ± 1.6*×*10^7^ transcripts) compared to the subsurface layer (9.4*×*10^5^ ± 5.2*×*10^5^ transcripts) of the lowlands.

Although gene abundance of *nosZ* in the subsurface was ~7 times larger in the montane forest (3.5*×*10^6^ ± 1.9*×*10^6^ transcripts) compared to the lowlands (5.0*×*10^5^ ± 4.3*×*10^5^ transcripts), this trend could not be observed in gene expression (Fig. 4i, j).

*16* *S* was 1.8 orders of magnitude more abundant and 1.6 orders of magnitude more expressed in the subsurface of the montane forest (2.2*×*10^10^ ± 1.1*×*10^10^ copies; 6.8*×*10^8^ ± 5.3*×*10^8^ transcripts, respectively) compared to the lowlands (3.7*×*10^8^ ± 3.0*×*10^8^ copies; 1.8*×*10^7^ ± 1.7*×*10^7^ transcripts, respectively).

## Discussion

### N_2_O fluxes

N_2_O fluxes in the lowlands showed little variation over the year. This is likely due to the lack of a distinct dry season, as indicated by constant WFPS at various depths. In the montane forest, however, WFPS decreased significantly during the dry season from July to August, resulting in a proportional decrease of N_2_O fluxes. Despite differences in temporal behavior, the N_2_O fluxes were similar at both sites (0.97 ± 0.79 kg N ha^−1^ year^−1^ in the lowlands and 0.88 ± 0.97 kg N ha^−1^ year^−1^ in the montane forest) but were lower than measurements in the Ankasa lowland forest in Ghana (2.30 kg N ha^−1^ year^−1^) and in the African montane forest in the Republic of Congo (2.91 kg N ha^−1^ year^−1^ [15]) and in Kenya (2.56 kg N ha^−1^ year^−1^ [14]). Furthermore, the N_2_O fluxes at our two study sites were at the lower end of fluxes reported in the Neotropics (1.15 to 6.53 kg N ha^−1^ year^−1^ [59–64]), Australian tropical forests (0.97 to 7.42 kg N ha^−1^ year^−1^ [65, 66]) and Indonesian forests (1.24 kg N ha^−1^ year^−1^ [67]). These low N_2_O fluxes can also be indirectly confirmed by the generally low N_2_O concentrations across soil depths (and time) at both sites. This study shows that the Congo Basin is unlikely to have contributed to the observed increase in N_2_O emissions coming from the African continent over the last two decades (as noted by Thompson et al. 2019 [68]). Instead, this increase might rather be attributed to increased livestock numbers and resulting manure accumulations in Sub-Saharan Africa; however, this pertains mostly to semi-arid and arid regions [69].

The Congo Basin is almost entirely covered by deeply weathered acidic Ferralsols [70]. Furthermore, a detailed vegetation map of the Congo Basin shows that lowland forests account for 90% and the montane forests for 2.6% of the total dense forest area present in the Congo Basin [71]. Thus, the dominance of Ferralsols and the selected forest types (lowland and montane forest) in the Congo Basin strengthen our confidence in our results being representative of large parts of the Congo Basin.

Numerous abiotic factors such as soil environmental conditions (temperature and WFPS) and physicochemical soil properties (texture, pH, C, N, Mn, Fe) have been reported to affect the production and consumption ratio of N_2_O (given by N_2_O/(N_2_ + N_2_O)) in soils [17, 22, 41]. However, despite significant differences in soil conditions and properties at the two forest sites, the mean N_2_O fluxes at both sites were similar, suggesting a multitude of controls on soil N_2_O fluxes in these systems. As a key factor, WFPS indirectly regulates the contribution of aerobic and anaerobic N_2_O production in terrestrial ecosystems through the availability of O_2_ in soils [72]. Optimal WFPS conditions for N_2_O emissions are in the range of 70–80% [73]. Conditions measured at our two study sites were generally on the range of 30–60%, in all but the montane upper 20 cm during the wet season (Fig. 3a, b). One may therefore expect aerobic processes such as nitrification to have an increased dominance. A WFPS around 60% further results in a product ratio of N_2_O/NO below 1.0 [72]. Generally, a lower WFPS may reduce denitrification and could explain the low N_2_O fluxes observed in both forests, when compared to other tropical forests [72]. In the montane forest, a higher clay content leading to a relatively larger WFPS, combined with a higher soil C content could provide more microsites for denitrifying communities, reducing N_2_O. Despite higher temperatures, N_2_O emissions in the lowlands were similar to in the montane forest, which could be explained by the sandy texture in the lowlands leading to lower WFPS and thus limiting denitrification.

In the absence of O_2_, DNRA is thermodynamically favored over denitrification under a high C:NO_3_^−^ ratio [18, 74]. In a humic tropical forest soil, rates of DNRA were found to be three times greater than the combined N_2_O and N_2_ fluxes from nitrification and denitrification [23]. Therefore, DNRA might have contributed to the overall N_2_O emissions in these forests. Emerging evidence further suggests that abiotic processes such as chemodenitrification [75] and the chemical decomposition of hydroxylamine [76] could be important N_2_O production processes in conditions of low pH and high Mn or Fe content. However, these ideal conditions were only found in the lowlands, where the pH was ~4 and Fe concentrations were relatively high (9–10 mg g^−1^ soil).

Despite different soil C and N contents in the lowlands and the montane forest, ∆^15^N values (i.e., the relative differences in isotopic signature (δ^15^N) between source (leaves) and sinks (litter and soil layers; Fig. 2) in the soil and litter of both forests were found to be similar, a result which suggests that both forests have similar N-cycle dynamics. This confirms a previous observation made at the same sites [5]. However, due to the different climatic conditions and the elevation effect a direct comparison of soil δ^15^N between lowland and montane forests is rather difficult [77]. Moreover, the lower N and larger soil C/N ratio found in the lowlands compared to the montane forest contradict the general paradigm of high soil N availability and thus an open N cycle.

### Indications of N_2_O reduction

**Fig. 5:**
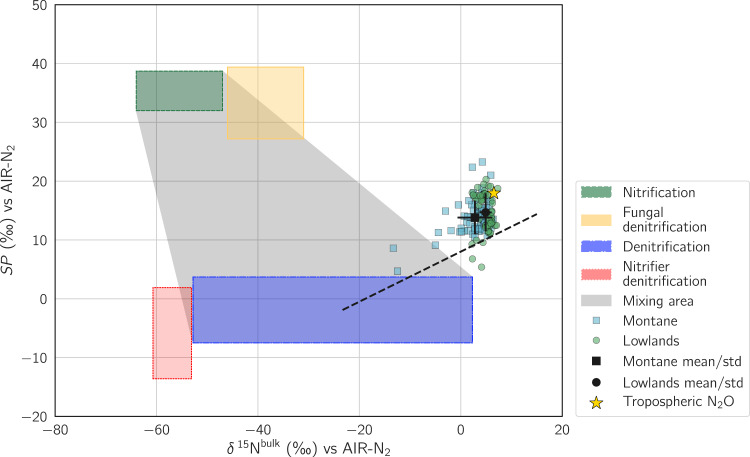
Isotope map using *SP* and *δ*^15^N^bulk^. A detailed description and discussion of end-member values for nitrification, fungal denitrification, denitrification, nitrifier denitrification, and theoretical reduction line (dashed line) can be found in Yu et al. 2020 [50] and references therein. Chemical denitrification is not included in the figure given large variations in observed values corresponding to different environments and substrates. Individual measurements are shown as green dots (lowlands) and blue squares (montane forest), respectively. Mean values and standard deviation at each site and over the entire measurement period across all depths are indicated in black. Average isotopic composition of tropospheric N_2_O (δ^15^N^bulk^, *SP*, δ^18^O [114]) is indicated by a yellow star. Mixing between nitrification and denitrification is shown in gray.

Soil pore N_2_O isotope measurements across a 1 m depth profile (*δ*^15^N^bulk^ = 4.1 ± 1.9‰, *SP* = 14.3 ± 2.9‰) in this study were in very good agreement with values measured across a 2 m depth profile in tropical forest soils of Panama (*δ*^15^N^bulk^ = 4.2‰, *SP* = 16.2‰ [61]). However, subsurface *δ*^15^N^bulk^ values measured in the Congo Basin were more ^15^N enriched compared to *δ*^15^N^bulk^ measurements of emitted surface N_2_O from primary forests of the Amazon (*−*12.3‰ [78], *−*18.0‰ [79]) and Costa Rica (*−*26.9 to *−*7.4‰ [73, 80]). Nevertheless, relying purely on *δ*^15^N^bulk^ for comparison and as a means to investigate N_2_O production and consumption pathways is challenging, especially when the isotopic composition of the precursor is unavailable [46]. In order to overcome this limitation and to determine the microbial origin of N_2_O, an isotope mapping approach was applied to further investigate our N_2_O isotope data, using the relationship between *δ*^15^N^bulk^ and *SP* (Fig. 5). Such mapping approaches have previously been applied in numerous studies [49, 56, 81–83] with a detailed summary in the recent review of Yu et al. 2020 [48], which includes updated end-member values (δ^18^O, *SP*, or δ^15^N^bulk^) of N_2_O produced from bacterial nitrification, fungal denitrification, denitrification, and nitrifier denitrification.

The application of the mapping approach revealed the likely dominance of biotic denitrification as the main N_2_O production pathway, corroborating observations made in other tropical forest soils [73, 84, 85].

If the N_2_O isotopic composition would be purely the result of mixing of N_2_O derived from nitrification and denitrification, one would expect the measured points to fall within the gray shaded area of the isomap (Fig. 5). However, in our dataset the observed values showed an enrichment relative to the mixing area, which could be explained by the isotope effect occurring during the reduction of N_2_O to N_2_. During N_2_O reduction, microorganisms favor lighter isotopes; thus, if a large proportion of N_2_O is subsequently reduced to N_2_, the residual N_2_O pool becomes relatively enriched, resulting in a simultaneous increase of *δ*^15^N^bulk^ and *SP* [86]. The occurrence of N_2_O reduction could explain the observation of relatively low soil N_2_O pore gas concentrations and relatively small surface N_2_O fluxes.

Nevertheless, although the dual mapping approach points towards reduction being an important process in these soils, an influence of atmospheric admixture [87, 88] or diffusive isotope effects [89] on observed *δ*^15^N^bulk^ and *SP* cannot be excluded.

In contrast, abiotic pathways such as the chemical decomposition of hydroxylamine are unlikely to contribute significantly to N_2_O emissions, given that *δ*^15^N^bulk^ and *SP* values from the emitted N_2_O measured in this study far exceed the range of values obtained from the literature ([42] and references therein).

Finally, the alternative use of *δ*^18^O and *SP* isomaps as compared to *δ*^15^N^bulk^ and *SP* isomaps has been suggested as a means to quantitatively determine N_2_ reduction [49]. This method shows some promise, since in contrast to *δ*^15^N^bulk^, *δ*^18^O is less subject to fractionation during microbial N_2_O production, as most of the oxygen in the N_2_O molecule originates from an exchange with water. Soil oxygen has a rather stable isotopic composition and the exchange with water exhibits a constant fractionation factor [49]. Nevertheless, due to the potential influence of factors such as admixture of atmospheric N_2_O with soil pore N_2_O, diffusive isotope effects, or the lack of pre-cursor isotopic information, quantitative reduction estimates would lead to erroneous results, especially when dealing with very low soil N_2_O concentrations.

Complementary to the isotopic analysis, the abundance and expression of N-cycling marker genes have been used to link the microbial community to N_2_O emissions [17, 22]. RNA typically has a short half-life in the environment, and its measurement can therefore provide an accurate snapshot of gene expression at the time of sampling. Correlating these measurements with physicochemical conditions can provide further insight into the drivers of denitrification [90]. *NirK* and *nirS* are responsible for catalyzing the reduction of NO_2_^–^ to NO (an important precursor of N_2_O), and their high abundance and expression across the soil layers at both sites indicates the potential of large production of N_2_O across the Congo Basin [91, 92]. However, despite the significant abundance and expression of *nirK* and *nirS*, measured N_2_O emissions were low. The abundance of the *nosZ* gene has been shown to negatively correlate with N_2_O emissions [92] since it encodes for the enzyme catalyzing the reduction of N_2_O to N_2_ [35]. A significant abundance and expression of *nosZ* across the soil layer (0–20 cm) at both sites indicate the enzymatic potential of N_2_O reduction, which could be an important microbial process in these forests.

Although functional gene analysis provides a powerful tool for the study of production and consumption processes in soils, some limitations to its application remain. Nucleotide sequences of current primers for functional genes are unable to capture the entire diversity of the target, for instance the fungal community. Additional uncertainties result from the use of *nosZ* primers only targeting the *nosZ* type I gene, as was done in this study, while the *nosZ* type II gene has been shown to be equally abundant and thereby more than doubling the known extent of overall N_2_O-reducers in the environment [93]. Furthermore, research has struggled to detect functional gene transcription activity under field conditions including that for denitrification genes [94].

The reduction of N_2_O to N_2_ is generally believed to decrease with increased acidity, since the N_2_O reductase *nosZ* is inhibited at low pH [95]. A negative correlation between the (N_2_O/(N_2_ + N_2_O)) product ratio and pH for pure cultures and microbial communities extracted from soil was confirmed by Bakken et al. 2012 [96]. Similar correlations have been observed in forest soils as well [97, 98]. Other studies, however, found no effect of pH on the transcription of *nosZ* [99, 100]. Similarly, we found that pH differences between the more acidic lowland soils compared to montane forest soils only minimally affected *nosZ* abundance or expression (Fig. 4) and did not affect N_2_O flux rates (Fig. 3).

A focus on *nosZ* gene bearing communities provided valuable information about the consumption of N_2_O in these forest soils, but a combination of separate primer sets for *nosZ* clade I and II for a variety of denitrifying communities including G^+^ denitrifiers hold great potential to further explain the microbial dynamics underlying our observed N_2_O fluxes.

### Implications for forest N budget

Regional N budgets suggest excessive N cycling in tropical forest soils of the Congo Basin mainly due to large atmospheric N deposition rates and as shown by a downregulation of biological N fixation in mature forests [3–5]. In contrast to many Neotropical forests, estimated N budgets of central African forests exhibit a higher apparent N input than N output [4]. High atmospheric N inputs are mainly derived from intensive seasonal biomass burning (18.2 kg N ha^−1^ year^−1^) and enter the system via wet deposition, with evidence of substantial extra input through dry deposition [4]. These large inputs are only partially balanced by hydrological dissolved nitrogen losses through soil leaching and streams (7.3 kg N ha^−1^ year^−1^), which suggests a missing N sink of at least 11.9 kg N ha^−1^ year^−1^ on a catchment scale [5]. The observation of an N saturated rather than an N limited ecosystem in central African forests is further supported by a study of the Nyungwe tropical montane forest in Rwanda [101].

Evidence of substantial gaseous N-losses (NO, N_2_O, N_2_) via denitrification were revealed in humid tropical forest soils of Hawaii [85], Southern China [84], and Costa Rica [102] using isotopic mass balances and isotopic modeling approaches. Gaseous N-losses were of similar magnitude and ranged between 2.0 and 9.8 kg N ha^−1^ year^−1^. However, observed N_2_O fluxes in the Congo Basin were low (0.9 kg N ha^−1^ year^−1^) and although isotopic and genetic data suggest an enzymatic potential for reduction of N_2_O to N_2_, additional N_2_-losses are likely unable to close the N budget of at least 11.9 N ha^−1^ year^−1^. The application of estimated median N_2_O/(N_2_O + N_2_) ratios for upland soils (0.49 [103] and 0.12 [104]) would result in N_2_-losses between 1.0 and 7.1 kg N ha^−1^ year^−1^ leading to combined N_2_O + N_2_ emissions of 2.0–8.1 kg N ha^−1^ year^−1^. However, estimated N_2_O/(N_2_O + N_2_) ratios represent a global median value, including different vegetation types (grassland, field, and forest types). In water-saturated tropical forest soils, N_2_ emissions will likely be at the higher end of the estimated spectrum. Applying N isotope budgets and process-based models, N_2_ emissions were estimated to be 4–5 times higher than N_2_O in old tropical forests [105, 106]. Indications for complete denitrification as a potential significant loss process was further found in a montane forest of Puerto Rico, where 45% of added ^15^NO_3_^−^ tracer could not be accounted for by plant and microbial uptake, leaching, DNRA or N_2_O fluxes [107]. Nevertheless, even maximal gaseous N-losses observed in Southern China (9.8 kg N ha^−1^ year^−1^, [84]) would be unable to close the N budget in central African forests. The contribution of high Fe concentrations in soils of the Congo Basin (Table S1) could fuel the production of N_2_ via anaerobic ammonium oxidation coupled to iron reduction (Feammox), short-circuiting the nitrogen cycle [108]. Feammox has been observed in tropical forest soils with iron concentrations of 6.2 ± 0.2 mg Fe per gram soil over a wide pH range, resulting in gaseous N-losses with rates comparable to denitrification [108]. As such, Feammox could be a potential alternative N-loss process in these forests, reducing N_2_O emissions if NH_4_^+^ is oxidized directly to N_2_. Especially in the montane forest, where Fe concentrations were ~10 times higher than in the lowland forest, this pathway could have contributed to the observed low N_2_O emissions. Nevertheless, due to the lack of accurate *in-situ* measurement techniques to study abiotic processes in the environment, the contribution of these processes is speculative and remains unknown. A further contributor to the missing N sink could be NO emissions. In Queensland Australia, NO emissions were observed to be ~8 times larger than N_2_O (~3.5 kg N ha^−1^ year^−1^ [109]) and NO emissions of old-growth tropical forest soils ranged between 0.1 and 7.9 kg N ha^−1^ year^−1^ ([110] and references therein).

In addition to gaseous and dissolved N-losses, N could leave the system via streams bound to organic matter as particulate organic Nitrogen (PON). This export is especially relevant in steep mountainous catchments that are prone to physical soil erosion [2, 111, 112]. Large PON exports on the Osa Peninsula, Costa Rica were mainly related to soil erosion during storm events and exceeded the dissolved forms at 14.6 kg N ha^−1^ year^−1^ [2]. With the use of a global sediment generation model, it was concluded that PON erosion may be an important N-loss process in geomorphologically active tropical landscapes [2]. However, our knowledge of transport mechanisms and the magnitude of PON exports via rivers in tropical landscapes is still very limited.

Low N_2_O fluxes in combination with the estimated N_2_ contributions shown in this study emphasize the imbalance of the N budget in central African forests, previously pointed out by Bauters et al. 2019 [5]. Future research is suggested to focus on NO and N_2_ gaseous N-losses, with a particular focus on N_2_ reduction and Feammox (in iron-rich soils). Furthermore, catchment-wide N budgets should include hydrological PON-losses. The importance of storm events as episodic mobilization agents of soil particulate organic matter in tropical landscapes has been pointed out by Hoover and Mackenzie 2009 [113]. As such, PON assessments should be coupled with hydrological monitoring to investigate the contribution of event-driven PON-losses.

## Conclusion

In this study, we investigated N_2_O fluxes over the course of a year in two different tropical forest ecosystems (lowlands and montane forest) of the Congo Basin. In order to understand spatiotemporal variations of N_2_O emissions we combined measurements of N_2_O isotopic signatures along soil profiles, with qPCR-derived gene abundance and expression data of nitrifying and denitrifying gene-bearing communities.

Our results show that the magnitude of N_2_O emissions at our two study sites is rather low compared to pantropical sites. Both the isotopic composition of soil pore N_2_O and the abundance and expression of the gene *nosZ* at the sites suggest that microbial reduction of N_2_O to N_2_ in the soil could partially explain these low N_2_O fluxes. However, even combined gaseous N-losses (N_2_O, N_2_) are unlikely to account for the missing N-sink observed in these forests. This suggests that alternative N-loss processes such as NO or N_2_ via Feammox as well as hydrological PON-losses could be significant in these forests. Improved analytical methods as well as constraining end-member values of microbial processes will hopefully facilitate the estimation of gaseous N-losses (N_2_, NO) in the future. In addition, investigations of catchment-wide processes including event-driven hydrological PON-losses could further advance our understanding of N-cycling in the Congo Basin. The dataset presented in this study is but a first step towards this understanding, and shows that there is an urgent need for further field measurements of central African forests.

## Supplementary information

Supplement

## References

[1] Vitousek PM, Sanford RL (1986). Nutrient cycling in moist tropical forest. Annu Rev Ecol Syst.

[2] Taylor PG, Wieder WR, Weintraub S, Cohen S, Cleveland CC, Townsend AR (2015). Organic forms dominate hydrologic nitrogen export from a lowland tropical watershed. Ecology..

[3] Bauters M, Mapenzi N, Kearsley E, Vanlauwe B, Boeckx P (2016). Facultative nitrogen fixation by legumes in the central Congo basin is downregulated during late successional stages. Biotropica..

[4] Bauters M, Drake TW, Verbeeck H, Bodé S, Hervé-Fernández P, Zito P (2018). High fire-derived nitrogen deposition on central African forests. Proc Natl Acad Sci.

[5] Bauters M, Verbeeck H, Rütting T, Barthel M, Bazirake Mujinya B, Bamba F (2019). Contrasting nitrogen fluxes in African tropical forests of the Congo Basin. Ecol Monogr.

[6] Brookshire ENJ, Gerber S, Menge DNL, Hedin LO (2012). Large losses of inorganic nitrogen from tropical rainforests suggest a lack of nitrogen limitation. Ecol Lett.

[7] Brookshire EJ, Thomas SA (2013). Ecosystem consequences of tree monodominance for nitrogen cycling in lowland tropical forest. PLOS ONE..

[8] Hedin LO, Brookshire ENJ, Menge DNL, Barron AR (2009). The nitrogen paradox in tropical forest ecosystems. Annu Rev Ecol, Evolution, Syst.

[9] Ipcc. Climate Change 2014: Synthesis Report. Contribution of Working Groups I, II and III to the Fifth Assessment Report of the Intergovernmental Panel on Climate Change. 2014.

[10] Ravishankara AR, Daniel JS, Portmann RW (2009). Nitrous oxide (N_2_O): The dominant ozone-depleting substance emitted in the 21st century. Science.

[11] Syakila A, Kroeze C (2011). The global nitrous oxide budget revisited. Greenh Gas Meas Manag.

[12] Werner C, Butterbach‐Bahl K, Haas E, Hickler T, Kiese R (2007). A global inventory of N_2_O emissions from tropical rainforest soils using a detailed biogeochemical model. Global Biogeochemical Cycles..

[13] Bai E, Houlton BZ, Wang YP (2012). Isotopic identification of nitrogen hotspots across natural terrestrial ecosystems. Biogeosciences.

[14] Castaldi S, Bertolini T, Valente A, Chiti T, Valentini R (2013). Nitrous oxide emissions from soil of an African rain forest in Ghana. Biogeosciences.

[15] Serca D, Delmas R, Jambert C, Labroue L (1994). Emissions of nitrogen oxides from equatorial rain forest in central Africa. Tellus B: Chem Phys Meteorol.

[16] Mayaux P, Pekel JF, Desclée B, Donnay F, Lupi A, Achard F (2013). State and evolution of the African rainforests between 1990 and 2010. Philosophical Transactions of the Royal Society B: Biological Sciences.

[17] Baggs EM (2008). A review of stable isotope techniques for N_2_O source partitioning in soils: recent progress, remaining challenges and future considerations. Rapid Commun Mass Spectrom.

[18] Tiedje JM, Ecology of denitrification and dissimilatory nitrate reduction to ammonium. Methods of Soil. Anal: Part 2 Chemical and Microbiological Properties. 1988;717:179–244.

[19] Tiedje JM (1988). Ecology of denitrification and dissimilatory nitrate reduction to ammonium. Biology of anaerobic microorganisms..

[20] Kool DM, Dolfing J, Wrage N, Van Groenigen JW (2011). Nitrifier denitrification as a distinct and significant source of nitrous oxide from soil. Soil Biol Biochem.

[21] Wrage N, Velthof GL, van Beusichem ML, Oenema O (2001). Role of nitrifier denitrification in the production of nitrous oxide. Soil Biol Biochem.

[22] Butterbach-Bahl K, Baggs EM, Dannenmann M, Kiese R, Zechmeister-Boltenstern S (2013). Nitrous oxide emissions from soils: how well do we understand the processes and their controls?. Philos Trans R Soc B: Biol Sci.

[23] Silver WL, Herman DJ, Firestone MK (2001). Dissimilatory nitrate reduction to ammonium in upland tropical forest soils. Ecology.

[24] Pandey CB, Kumar U, Kaviraj M, Minick KJ, Mishra AK, Singh JS (2020). DNRA: A short-circuit in biological N-cycling to conserve nitrogen in terrestrial ecosystems. Science of the Total Environment..

[25] Leininger S, Urich T, Schloter M, Schwark L, Qi J, Nicol GW (2006). Archaea predominate among ammonia-oxidizing prokaryotes in soils. Nature.

[26] Rotthauwe JH, Witzel KP, Liesack W (1997). The ammonia monooxygenase structural gene *amoA* as a functional marker: molecular fine-scale analysis of natural ammonia-oxidizing populations. Appl Environ Microbiol.

[27] Schauss K, Focks A, Leininger S, Kotzerke A, Heuer H, Thiele-Bruhn S (2009). Dynamics and functional relevance of ammonia-oxidizing archaea in two agricultural soils. Environ Microbiol.

[28] Hayatsu M, Tago K, Saito M (2008). Various players in the nitrogen cycle: Diversity and functions of the microorganisms involved in nitrification and denitrification. Soil Sci Plant Nutr.

[29] Henry S, Baudoin E, López-Gutiérrez JC, Martin-Laurent F, Brauman A, Philippot L (2004). Quantification of denitrifying bacteria in soils by *nirK* gene targeted real-time PCR. J Microbiological Methods.

[30] Kandeler E, Deiglmayr K, Tscherko D, Bru D, Philippot L (2006). Abundance of *narG*, *nirS*, *nirK*, and *nosZ* genes of denitrifying bacteria during primary successions of a glacier foreland. Appl Environ Microbiol.

[31] Throbäck IN, Enwall K, Jarvis Å, Hallin S (2004). Reassessing PCR primers targeting *nirS*, *nirK* and *nosZ* genes for community surveys of denitrifying bacteria with DGGE. FEMS Microbiol Ecol.

[32] Jones CM, Graf DRH, Bru D, Philippot L, Hallin S (2013). The unaccounted yet abundant nitrous oxide-reducing microbial community: a potential nitrous oxide sink. ISME J.

[33] Orellana LH, Rodriguez-R LM, Higgins S, Chee-Sanford JC, Sanford RA, Ritalahti KM (2014). Detecting nitrous oxide reductase (*nosZ*) genes in soil metagenomes: Method development and implications for the nitrogen cycle. mBio..

[34] Sanford RA, Wagner DD, Wu Q, Chee-Sanford JC, Thomas SH, Cruz-García C (2012). Unexpected nondenitrifier nitrous oxide reductase gene diversity and abundance in soils. Proc Natl Acad Sci.

[35] Henry S, Bru D, Stres B, Hallet S, Philippot L (2006). Quantitative detection of the *nosZ* gene, encoding nitrous oxide reductase, and comparison of the abundances of *16S* rRNA, *narG*, *nirK*, and *nosZ* genes in soils. Appl Environ Microbiol.

[36] Graf DR, Jones CM, Hallin S (2014). Intergenomic comparisons highlight modularity of the denitrification pathway and underpin the importance of community structure for N_2_O emissions. PlOS ONE..

[37] Philippot L, Hallin S, Schloter M (2007). Ecology of denitrifying prokaryotes in agricultural soil. Adv Agron.

[38] Mothapo N, Chen H, Cubeta MA, Grossman JM, Fuller F, Shi W (2015). Phylogenetic, taxonomic and functional diversity of fungal denitrifiers and associated N_2_O production efficacy. Soil Biol Biochem.

[39] Bremner JM (1997). Sources of nitrous oxide in soils. Nutrient Cycl Agroecosyst.

[40] Heil J, Liu S, Vereecken H, Brüggemann N (2015). Abiotic nitrous oxide production from hydroxylamine in soils and their dependence on soil properties. Soil Biol Biochem.

[41] Firestone MK, Davidson EA (1989). Microbiological basis of NO and N_2_O production and consumption in soil. Exchange of trace gases between terrestrial ecosystems and the atmosphere..

[42] Denk TRA, Mohn J, Decock C, Lewicka-Szczebak D, Harris E, Butterbach-Bahl K (2017). The nitrogen cycle: A review of isotope effects and isotope modeling approaches. Soil Biol Biochem.

[43] Pérez T, Trumbore SE, Tyler SC, Matson PA, Ortiz-Monasterio I, Rahn T (2001). Identifying the agricultural imprint on the global N_2_O budget using stable isotopes. J Geophys Res: Atmospheres.

[44] Brenninkmeijer Rockmann (1999). Mass spectrometry of the intramolecular nitrogen isotope distribution of environmental nitrous oxide using fragment-ion analysis. Rapid Commun Mass Spectrom: RCM.

[45] Toyoda S, Yoshida N (1999). Determination of nitrogen isotopomers of nitrous oxide on a modified isotope ratio mass spectrometer. Anal Chem.

[46] Decock C, Six J (2013). How reliable is the intramolecular distribution of ^15^N in N_2_O to source partition N_2_O emitted from soil?. Soil Biol Biochem.

[47] Yang H, Gandhi H, Ostrom NE, Hegg EL (2014). Isotopic fractionation by a fungal *P450* nitric oxide reductase during the production of N_2_O. Environ Sci Technol.

[48] Yu L, Harris E, Lewicka‐Szczebak D, Barthel M, Blomberg MR, Harris SJ (2020). What can we learn from N_2_O isotope data? - Analytics, processes and modelling. Rapid Commun Mass Spectrom.

[49] Lewicka-Szczebak D, Augustin J, Giesemann A, Well R (2017). Quantifying N_2_O reduction to N_2_ based on N_2_O isotopocules – validation with independent methods (helium incubation and ^15^N gas flux method). Biogeosciences.

[50] Peh KS, Sonké B, Lloyd J, Quesada CA, Lewis SL (2011). Soil does not explain monodominance in a Central African tropical forest. PLOS ONE..

[51] Van Ranst E, Baert G, Ngongo M, Mafuka P. Carte pédologique de Yangambi, planchette 2: Yangambi, échelle 1: 50.000. UGent; Hogent; UNILU; UNIKIN; 2010.

[52] Imani G, Zapfack L, Kalume J, Riera B, Cirimwami L, Boyemba F (2016). Woody vegetation groups and diversity along the altitudinal gradient in mountain forest: case study of Kahuzi-Biega National Park and its surroundings. RD Congo. Journal of Biodiversity and Environmental Sciences..

[53] Sparks DL, Page AL, Helmke PA, Loeppert RH, editors. Methods of soil analysis, part 3: Chemical methods. John Wiley & Sons; 2020 Jan 22.

[54] Brooks PD, Geilmann H, Werner RA, Brand WA (2003). Improved precision of coupled δ^13^C and δ^15^N measurements from single samples using an elemental analyzer/isotope ratio mass spectrometer combination with a post-column six-port valve and selective CO_2_ trapping; improved halide robustness of the combustion reactor using CeO_2_. Rapid Commun Mass Spectrom.

[55] Werner RA, Brand WA (2001). Referencing strategies and techniques in stable isotope ratio analysis. Rapid Commun Mass Spectrom.

[56] Verhoeven E, Barthel M, Yu L, Celi L, Said-Pullicino D, Sleutel S (2019). Early season N_2_O emissions under variable water management in rice systems: source-partitioning emissions using isotope ratios along a depth profile. Biogeosciences..

[57] Mohn J, Wolf B, Toyoda S, Lin CT, Liang MC, Brüggemann N, et al. Interlaboratory assessment of nitrous oxide isotopomer analysis by isotope ratio mass spectrometry and laser spectroscopy: current status and perspectives. Rapid communications in mass spectrometry. 2014;28:1995–2007. 10.1002/rcm.6982 25132300

[58] Suzuki MT, Taylor LT, DeLong EF (2000). Quantitative analysis of small-subunit rRNA genes in mixed microbial populations via 5′-nuclease assays. Appl Environ Microbiol.

[59] Davidson EA, Nepstad DC, Ishida FY, Brando PM (2008). Effects of an experimental drought and recovery on soil emissions of carbon dioxide, methane, nitrous oxide, and nitric oxide in a moist tropical forest. Glob Change Biol.

[60] Keller M, Varner R, Dias JD, Silva H, Crill P, de Oliveira RC (2005). Soil–atmosphere exchange of nitrous oxide, nitric oxide, methane, and carbon dioxide in logged and undisturbed forest in the Tapajos National Forest, Brazil. Earth Interact.

[61] Koehler B, Corre MD, Steger K, Well R, Zehe E, Sueta JP (2012). An in-depth look into a tropical lowland forest soil: nitrogen-addition effects on the contents of N_2_O, CO_2_ and CH_4_ and N_2_O isotopic signatures down to 2m depth. Biogeochemistry..

[62] Maddock JEL, dos Santos MBP, Prata KR (2001). Nitrous oxide emission from soil of the Mata Atlantica, Rio de Janeiro State, Brazil. J Geophys Res: Atmospheres.

[63] Melillo JM, Steudler PA, Feigl BJ, Neill C, Garcia D, Piccolo MC (2001). Nitrous oxide emissions from forests and pastures of various ages in the Brazilian Amazon. J Geophys Res: Atmospheres.

[64] Nepstad DC, Moutinho P, Dias-Filho MB, Davidson E, Cardinot G, Markewitz D (2002). The effects of partial throughfall exclusion on canopy processes, aboveground production, and biogeochemistry of an Amazon forest. J Geophys Res: Atmospheres.

[65] Breuer L, Papen H, Butterbach-Bahl K (2000). N_2_O emission from tropical forest soils of Australia. J Geophys Res: Atmospheres.

[66] Kiese R, Butterbach-Bahl K (2002). N_2_O and CO_2_ emissions from three different tropical forest sites in the wet tropics of Queensland, Australia. Soil Biol Biochem.

[67] Verchot LV, Hutabarat L, Hairiah K, Van, Noordwijk M (2006). Nitrogen availability and soil N_2_O emissions following conversion of forests to coffee in southern Sumatra. Global Biogeochemical Cycles..

[68] Thompson R, Lassaletta L, Patra P, Wilson C, Wells K, Gressent A (2019). Acceleration of global N_2_O emissions seen from two decades of atmospheric inversion. Nat Clim Change.

[69] Butterbach-Bahl K, Gettel G, Kiese R, Fuchs K, Werner C, Rahimi J (2020). Livestock enclosures in drylands of Sub-Saharan Africa are overlooked hotspots of N_2_O emissions. Nat Commun.

[70] Jones A, Breunig-Mafsen H, Brossard M, Dampha A, Deckers J, Dewitte O, et al. Soil Atlas of Africa. European Commission; 2013.

[71] Verhegghen A, Mayaux P, De Wasseige C, Defourny P (2012). Mapping Congo Basin vegetation types from 300 m and 1 km multi-sensor time series for carbon stocks and forest areas estimation. Biogeosciences..

[72] Davidson EA. Soil water content and the ratio of nitrous oxide to nitric oxide emitted from soil. In: Biogeochemistry of global change. Springer; 1993. pp. 369-86.

[73] Pérez T, Trumbore SE, Tyler SC, Davidson EA, Keller M, de Camargo PB (2000). Isotopic variability of N_2_O emissions from tropical forest soils. Glob Biogeochem Cycles.

[74] Strohm TO, Griffin B, Zumft WG, Schink B (2007). Growth yields in bacterial denitrification and nitrate ammonification. Appl Environ Microbiol.

[75] Heil J, Vereecken H, Brüggemann N (2016). A review of chemical reactions of nitrification intermediates and their role in nitrogen cycling and nitrogen trace gas formation in soil: Chemical reactions of nitrification intermediates in soil. Eur J Soil Sci.

[76] Liu S, Berns AE, Vereecken H, Wu D, Brüggemann N (2017). Interactive effects of MnO_2_, organic matter and pH on abiotic formation of N_2_O from hydroxylamine in artificial soil mixtures. Sci Rep.

[77] Bauters M, Verbeeck H, Demol M, Bruneel S, Taveirne C, Heyden DV (2017). Parallel functional and stoichiometric trait shifts in South American and African forest communities with elevation. Biogeosciences..

[78] Pérez T, Garcia-Montiel D, Trumbore S, Tyler S, de Camargo P, Moreira M (2006). Nitrous oxide nitrification and denitrification ^15^N enrichment factors from Amazon forest soils. Ecol Appl.

[79] Park S, Pérez T, Boering KA, Trumbore SE, Gil J, Marquina S (2011). Can N_2_O stable isotopes and isotopomers be useful tools to characterize sources and microbial pathways of N_2_O production and consumption in tropical soils?. Global Biogeochem Cycles..

[80] Kim KR, Craig H (1993). Nitrogen-15 and oxygen-18 characteristics of nitrous oxide: a global perspective. Science.

[81] Wu D, Well R, Càrdenas LM, Fuss R, Lewicka-Szczebak D, Reent KJ (2019). Quantifying N_2_O reduction to N_2_ during denitrification in soils via isotopic mapping approach: Model evaluation and uncertainty analysis. Environ Res.

[82] Ibraim E, Wolf B, Harris E, Gasche R, Wei J, Yu L (2019). Attribution of N_2_O sources in a grassland soil with laser spectroscopy based isotopocule analysis. Biogeosciences..

[83] Buchen C, Lewicka‐Szczebak D, Flessa H, Well R (2018). Estimating N_2_O processes during grassland renewal and grassland conversion to maize cropping using N_2_O isotopocules. Rapid Commun Mass Spectrom.

[84] Fang Y, Koba K, Makabe A, Takahashi C, Zhu W, Hayashi T (2015). Microbial denitrification dominates nitrate losses from forest ecosystems. Proc Natl Acad Sci.

[85] Houlton BZ, Sigman DM, Hedin LO (2006). Isotopic evidence for large gaseous nitrogen losses from tropical rainforests. Proc Natl Acad Sci.

[86] Well R, Kurganova I, de Gerenyu VL, Flessa H (2006). Isotopomer signatures of soil-emitted N_2_O under different moisture conditions—a microcosm study with arable loess soil. Soil Biol Biochem.

[87] Goldberg SD, Knorr K-H, Gebauer G (2008). N_2_O concentration and isotope signature along profiles provide deeper insight into the fate of N_2_O in soils. Isotopes Environ Health Stud.

[88] Snider DM, Venkiteswaran JJ, Schiff SL, Spoelstra J (2015). From the ground up: Global nitrous oxide sources are constrained by stable isotope values. PlOS ONE..

[89] Well R, Flessa H (2008). Isotope fractionation factors of N_2_O diffusion. Rapid Commun Mass Spectrom.

[90] Groffman PM, Altabet MA, Böhlke hJK, Butterbach-Bahl K, David MB, Firestone MK (2006). Methods for measuring denitrification: diverse approaches to a difficult problem. Ecol Appl.

[91] Morales SE, Cosart T, Holben WE (2010). Bacterial gene abundances as indicators of greenhouse gas emission in soils. ISME J.

[92] Rasche F, Knapp D, Kaiser C, Koranda M, Kitzler B, Zechmeister-Boltenstern S (2011). Seasonality and resource availability control bacterial and archaeal communities in soils of a temperate beech forest. ISME J.

[93] Jones CM, Graf DR, Bru D, Philippot L, Hallin S (2013). The unaccounted yet abundant nitrous oxide-reducing microbial community: a potential nitrous oxide sink. ISME J.

[94] Levy-Booth DJ, Prescott CE, Grayston SJ (2014). Microbial functional genes involved in nitrogen fixation, nitrification and denitrification in forest ecosystems. Soil Biol Biochem.

[95] Knowles R (1982). Denitrification. Microbiological Rev.

[96] Bakken LR, Bergaust L, Liu B, Frostegård A (2012). Regulation of denitrification at the cellular level: a clue to the understanding of N_2_O emissions from soils. Philos Trans R Soc B: Biol Sci.

[97] Liu B, Mørkved PT, Frostegård A, Bakken LR (2010). Denitrification gene pools, transcription and kinetics of NO, N_2_O and N_2_ production as affected by soil pH. FEMS Microbiol Ecol.

[98] Richardson D, Felgate H, Watmough N, Thomson A, Baggs E (2009). Mitigating release of the potent greenhouse gas N_2_O from the nitrogen cycle - could enzymic regulation hold the key?. Trends Biotechnol.

[99] Bergaust L, Mao Y, Bakken LR, Frostegård Å (2010). Denitrification response patterns during the transition to anoxic respiration and posttranscriptional effects of suboptimal pH on nitrogen oxide reductase in Paracoccus denitrificans. Appl Environ Microbiol.

[100] Henderson SL, Dandie CE, Patten CL, Zebarth BJ, Burton DL, Trevors JT (2010). Changes in denitrifier abundance, denitrification gene mRNA levels, nitrous oxide emissions, and denitrification in anoxic soil microcosms amended with glucose and plant residues. Appl Environ Microbiol.

[101] Rütting T, Cizungu Ntaboba L, Roobroeck D, Bauters M, Huygens D, Boeckx P (2015). Leaky nitrogen cycle in pristine African montane rainforest soil. Glob Biogeochemical Cycles.

[102] Soper FM, Taylor PG, Wieder WR, Weintraub SR, Cleveland CC, Porder S, et al. Modest gaseous nitrogen losses point to conservative nitrogen cycling in a lowland tropical forest watershed. Ecosystems. 2018;21:901–12.

[103] Schlesinger WH (2009). On the fate of anthropogenic nitrogen. Proc Natl Acad Sci.

[104] Scheer C, Fuchs K, Pelster DE, Butterbach-Bahl K (2020). Estimating global terrestrial denitrification from measured N_2_O:(N_2_O + N_2_) product ratios. Current Opinion in Environmental. Sustainability.

[105] Hedin LO, Vitousek PM, Matson PA (2003). Nutrient losses over four million years of tropical forest development. Ecology..

[106] Bai E, Houlton BZ (2009). Coupled isotopic and process‐based modeling of gaseous nitrogen losses from tropical rain forests. Global Biogeochemical Cycles.

[107] Templer PH, Silver WL, Pett-Ridge J, DeAngelis M, Firestone K (2008). MK. Plant and microbial controls on nitrogen retention and loss in a humid tropical forest. Ecology.

[108] Yang WH, Weber KA, Silver WL (2012). Nitrogen loss from soil through anaerobic ammonium oxidation coupled to iron reduction. Nat Geosci.

[109] Butterbach‐Bahl K, Kock M, Willibald G, Hewett B, Buhagiar S, Papen H (2004). Temporal variations of fluxes of NO, NO_2_, N_2_O, CO_2_, and CH_4_ in a tropical rain forest ecosystem. Global Biogeochem Cycles.

[110] Koehler B, Corre MD, Veldkamp E, Wullaert H, Wright SJ (2009). Immediate and long-term nitrogen oxide emissions from tropical forest soils exposed to elevated nitrogen input. Glob Change Biol.

[111] Kao S, Liu K (2000). Stable carbon and nitrogen isotope systematics in a human‐disturbed watershed (Lanyang‐Hsi) in Taiwan and the estimation of biogenic particulate organic carbon and nitrogen fluxes. Glob Biogeochem Cycles.

[112] Townsend-Small A, McClain ME, Hall B, Noguera JL, Llerena CA, Brandes JA (2008). Suspended sediments and organic matter in mountain headwaters of the Amazon River: Results from a 1-year time series study in the central Peruvian Andes. Geochimica et Cosmochimica Acta.

[113] Hoover D, Mackenzie F (2009). Fluvial fluxes of water, suspended particulate matter, and nutrients and potential impacts on tropical coastal water biogeochemistry: Oahu, Hawai’i. Aquat Geochem.

[114] Harris E, Henne S, Hüglin C, Zellweger C, Tuzson B, Ibraim E (2017). Tracking nitrous oxide emission processes at a suburban site with semicontinuous, in situ measurements of isotopic composition. Journal of Geophysical Research: Atmospheres..

