# Supplementary material for: In-depth analysis of N2O fluxes in tropical forest soils of the Congo Basin combining isotope and functional gene analysis
Source: ISME J. 2021 May 25;15(11):3357–74. doi: 10.1038/s41396-021-01004-x (PMC8528805; doi:10.1038/s41396-021-01004-x)
Supplement: Supplementary file 1 — Supplement [file 41396_2021_1004_MOESM1_ESM.pdf]

Supplement

Table S1. Micronutrients at the two forest sites (lowlands, montane) at the surface layer (0-5 cm) and the sub-surface layer (5-20 cm).

		Lowlands		Montane	
**Micronutrient**		**0-5 cm**	**5-20 cm**	**0-5 cm**	**5-20 cm**
Al	[mg/g soil]	9.77 ± 1.72	19.34 ± 1.86	69.58 ± 7.90	81.58 ± 2.48
Cu	[µg/g soil]	3.80 ± 0.49	3.77 ± 0.59	57.90 ± 6.55	68.25 ± 0.51
Mn	[mg/g soil]	0.04 ± 0.01	0.04 ± 0.00	2.73 ± 0.13	2.71 ± 0.65
Mg	[mg/g soil]	0.10 ± 0.02	0.08 ± 0.01	2.60 ± 0.36	2.22 ± 0.60
Na	[mg/g soil]	0.03 ± 0.00	0.02 ± 0.01	0.11 ± 0.01	0.11 ± 0.02
Pb	[µg/g soil]	22.5 ± 3.35	25.46 ± 4.06	170.55 ± 16.34	202.74 ± 16.14
Ca	[mg/g soil]	0.05 ± 0.01	0.03 ± 0.01	3.89 ± 0.49	1.11 ± 0.91
Fe	[mg/g soil]	9.44 ± 1.21	10.52 ± 1.01	48.26 ± 2.48	53.61 ± 2.01
Mn	[mg/g soil]	0.04 ± 0.01	0.04 ± 0.00	2.73 ± 0.13	2.71 ± 0.65
Ni	[µg/g soil]	2.16 ± 0.54	1.90 ± 0.43	106.14 ± 8.96	124.16 ± 3.38
S	[mg/g soil]	0.20 ± 0.02	0.12 ± 0.01	0.75 ± 0.09	0.61 ± 0.02
Cd	[µg/g soil]	1.23 ± 0.17	1.37 ± 0.18	11.04 ± 0.91	12.82 ± 1.23
K	[mg/g soil]	0.16 ± 0.01	0.14 ± 0.03	1.17 ± 0.02	1.11 ± 0.06
Mo	[mg/g soil]	0.64 ± 0.60	0.67 ± 0.37	1.62 ± 0.15	1.78 ± 0.18
P	[mg/g soil]	0.17 ± 0.02	0.15 ± 0.02	1.77 ± 0.36	1.71 ± 0.19
Zn	[µg/g soil]	36.23 ± 1.08	13.92 ± 3.47	124.14 ± 17.42	116.12 ± 19.17

Table S2. Master Mix composition and DNA/cDNA extracts used for the real-time qPCR. All amounts are provided per replicate.

Gene	Gene Specific Dyes	Primers	ddH_2O	DNA/cDNA	Reference
16S	10 µL TaqMan Universal PCR Master Mix<br>0.4 µL Fluorogenic probe TM1389 (200 nmol)<br>Applied Biosystems, Foster City, CA, USA	0.8 µL Forward Primer (800 nmol)<br>0.8 µL Reverse Primer (800 nmol)	4 µL	4 µL<br>1:100 diluted 1:20 diluted cDNA extract	(1)
amoA AOA	5 µL Kapa SYBR Fast Universal Master Mix<br>0.2 µL Reference dye Rox low (0.05 mol)<br>Kapa Biosystems, Wilmington, MA, USA	0.75 µL Forward Primer (0.375 mol)<br>0.75 µL Reverse Primer (0.375 mol)	2.3 µL	3 µL<br>1:20 diluted 1:20 diluted	(2, 3)
amoA AOB	5 µL Kapa SYBR Fast Universal Master Mix<br>0.2 µL reference dye Rox low (0.05 mol)<br>Kapa Biosystems, Wilmington, MA, USA	1 µL Forward Primer (0.5 mol)<br>1 µL Reverse Primer (0.5 mol)	1.8 µL	3 µL<br>1:20 diluted 1:20 diluted	(4)
nirK	12.5 µL ABI Power SYBR Green PCR Master Mix<br>Applied Biosystems, Foster City, CA, USA	0.5 µL Forward Primer (0.5 mol)<br>0.5 µL Reverse Primer (0.5 mol)	6.5 µL	5 µL<br>1:100 diluted 1:20 diluted	(5)
nirS	12.5 µL ABI Power SYBR Green PCR Master Mix<br>Applied Biosystems, Foster City, CA, USA	0.5 µL Forward Primer (0.5 mol)<br>0.5 µL Reverse Primer (0.5 mol)	6.5 µL	5 µL<br>1:50 diluted 1:20 diluted	(6, 7)
nosZ	10 µL ABI Power SYBR Green PCR Master Mix<br>Applied Biosystems, Foster City, CA, USA	0.8 µL Forward Primer (0.3 mol)<br>0.8 µL Reverse Primer (0.3 mol)	3.4 µL	5 µL<br>1:50 diluted 1:20 diluted	(8)

Table S3. Primers and PCR conditions used for real-time qPCR.

Gene	Primer	Primer sequence (5' – 3')	Thermal profile	Reference
16S	BACT1369F<br>PROK1492R<br>TM1389	CGGTGAATACGTTCYCGG<br>AAGGAGGTGATCCRGCCGC<br>CTTGTACACACCGCCCGTC	50 °C, 2 min, 95 °C, 10 min, 40 cycles: 95 °C, 15 s, 56 °C, 60 s	(1)
amoA AOA	amo19F<br>crenamo	ATGGTCTGGCTWAGACG<br>GCCATCCABCKRTANGTCCA	15 s at 95 °C, 40 cycles: 95 °C, 15 s 55 °C, 15 s, 72 °C, 31 s 95 °C, 15 s 60 °C, 30 s, 95 °C, 15 s	(2, 3)
amoA AOB	amoA1F<br>amoA2R	GGGGTTTCTACTGGTGGT<br>CCCCTCKGSAAAGCCTTCTTC	60 s at 95 °C, 40 cycles: 95 °C, 15 s 59.5 °C, 30 s, 72 °C, 30 s 95 °C, 60 s 55 °C, 60 s, 55 to 95 °C, 10 s	(4)
nirK	nirKc876<br>nirK1040	ATYGGCGGVCAYGGCGA<br>GCCTCGATAGRTTRTGGTT	95 °C, 30 s, 5 cycles: 95 °C, 15 s, 63<br>59 °C (–1 °C per cycle), 30 s, 72 °C<br>30 s, 30 cycles: 95 °C, 15 s, 63 °C, 30 s, 72 °C, 30 s	(10
nirS	nirSCd3aF<br>nirSR3cd	AACGYSAAGGARACSGG<br>GASTTCGGRTGSGTCTTSAYGAA	95 °C, 10 min, 35 cycles: 95 °C, 15 s, 63 °C, 30 s, 72 °C, 30 s, 80 °C, 30 s	(6, 7)
nosZ	nosZ2F<br>nosZ2R	CGCRACGGCAASAAGGTSMSSGT<br>ACAKRTGCAKSGCRTGGCAGA	95 °C, 10 min 6 cycles: 95 °C, 15 s<br>65 °C 30 s, 72 °C, 30 s, 40 cycles:<br>95 °C, 15 s 60 °C, 15 s, 72 °C, 30 s, 83 °C, 30 s	(8)

References

1. Suzuki MT, Taylor LT, DeLong EF. Quantitative Analysis of Small-Subunit rRNA Genes in Mixed Microbial Populations via 5′-Nuclease Assays. Applied and Environmental Microbiology. 2000;66(11):4605-14.
2. Leininger S, Urich T, Schloter M, Schwark L, Qi J, Nicol GW, et al. Archaea predominate among ammonia-oxidizing prokaryotes in soils. Nature. 2006;442(7104):806-9.
3. Schauss K, Focks A, Leininger S, Kotzerke A, Heuer H, Thiele-Bruhn S, et al. Dynamics and functional relevance of ammonia-oxidizing archaea in two agricultural soils. Environmental Microbiology. 2009;11(2):446-56.
4. Rotthauwe JH, Witzel KP, Liesack W. The ammonia monooxygenase structural gene amoA as a functional marker: molecular fine-scale analysis of natural ammonia-oxidizing populations. Applied and Environmental Microbiology. 1997;63(12):4704-12.
5. Henry S, Bru D, Stres B, Hallet S, Philippot L. Quantitative detection of the nosZ gene, encoding nitrous oxide reductase, and comparison of the abundances of 16S rRNA, narG, nirK, and nosZ genes in soils. Applied and Environmental Microbiology. 2006;72(8):5181-9.
6. Kandeler E, Deiglmayr K, Tscherko D, Bru D, Philippot L. Abundance of narG, nirS, nirK, and nosZ Genes of Denitrifying Bacteria during Primary Successions of a Glacier Foreland. Applied and Environmental Microbiology. 2006;72(9):5957-62.
7. Throbäck IN, Enwall K, Jarvis Å, Hallin S. Reassessing PCR primers targeting nirS, nirK and nosZ genes for community surveys of denitrifying bacteria with DGGE. FEMS Microbiology Ecology. 2004;49(3):401-17.
8. Henry S, Baudoin E, López-Gutiérrez JC, Martin-Laurent F, Brauman A, Philippot L. Quantification of denitrifying bacteria in soils by nirK gene targeted real-time PCR. Journal of Microbiological Methods. 2004;59(3):327-35.
